# Opportunistic assessment of bone mineral density in cystic fibrosis patients using ultra-low dose thoracic CT

**DOI:** 10.1007/s11657-025-01578-5

**Published:** 2025-07-12

**Authors:** Sahil Shet, Catherine Henry, Mairead O’Donnell, Eid Kakish, Muhammad Ghauri, Patrick O’Regan, Kevin Deasy, Hisham Ibrahim, Michael Maher, Barry Plant, David J. Ryan

**Affiliations:** 1 Department of Radiology, School of Medicine, University College Cork, Cork, Ireland; 2 Department of Radiology, Cork University Hospital, Cork, Ireland; 3 Department of Respiratory Medicine, Cork University Hospital, Wilton, Cork Ireland; 4 Department of Medicine, Cork University Hospital and University College Cork, Cork, Ireland; 5 School of Medicine, University College Cork, Cork, Ireland

**Keywords:** Bone mineral density, Cystic fibrosis, Morphomics

## Abstract

**Summary:**

In this study, we used routine ultra-low dose computed tomography scans of patients with cystic fibrosis to predict bone mineral density (BMD). A strong correlation was found between the attenuation of trabecular bone in thoracic vertebrae and the BMD in the proximal femur and lumbar spine as measured on DEXA.

**Purpose:**

Osteoporosis is a serious global health concern with millions of people affected worldwide. A particularly vulnerable cohort in developing osteoporosis are patients with cystic fibrosis (CF). Bone mineral density (BMD) is typically measured with dual energy X-ray absorptiometry (DEXA) scanning; however, this comes at a cost to the healthcare system and an exposure to ionising radiation. In our institution, patients with cystic fibrosis undergo routine ultra-low dose computed tomography (ULDCT) for monitoring of disease progression. The aim of this study was to assess the validity of estimating BMD using data derived from ULDCT scans.

**Methods:**

Adult CF patients were included if they had undergone a routine ULDCT scan within 12 months of a DEXA scan. Additionally, 100 non-CF patients with non-contrast standard dose CT scans were selected to act as the control group. Trabecular bone density (T-BD) at T4, T7 and T10 was measured on PACS in Hounsfield units (HU) and compared to DEXA scan results and a formula developed to the predict BMD.

**Results:**

Fifty-two female and 62 male patients were included with mean ages of 34.4 and 35.1 respectively. Moderately strong correlation was found between the T-BD and BMD of both the lumbar spine (*r* = 0.629, *p* < 0.001) and proximal femur (*r* = 0.649, *p* < 0.001). Receiver operator characteristic (ROC) curve analysis found a sensitivity and specificity of 0.700 and 0.714 respectively at predicting osteoporosis at T-BD of 193.33 HU or below.

**Conclusion:**

T-BD measured on ULDCT may be a valuable tool in the early identification of CF patients at risk of osteoporosis.

## Introduction

Low bone mineral density, of which osteoporosis is the most severe form, has become a serious global health concern over the last few decades [1]. Indeed, it is estimated that over 44 million people in the USA and approximately 200 million worldwide are suffering with osteoporosis with an approximate yearly cost of 25.3 billion dollars in the USA alone [2, 3]. As the ageing population continues to rise, so too does the prevalence of osteoporosis and consequently, fragility fractures. In Ireland, fracture admissions increased by 30% between 2000 and 2014 with a prediction that admissions will continue to rise over the next three decades [4].

Although there have been considerable advances in the treatment for established osteoporosis, unfortunately, diagnosis is commonly made following a fragility fracture which has already occurred, thus shifting the treatment focus to future fracture prevention [5, 6].

Several strategies for early diagnosis have been proposed including screening women over 65 and post-menopausal women under 65 with an increased risk of osteoporosis [7]. In addition, certain cohorts of patients, such as those with cystic fibrosis (CF), are also at an increased risk of osteoporosis and regular screening of these patients with DEXA scanning has been recommended [8]. As a consequence of improving treatment strategies for cystic fibrosis patients, the average life expectancy has increased significantly over the last four decades that resulted in rising numbers of patients with cystic fibrosis bone disease (CFBD) [9].

CFBD has a complex aetiology and the cause is likely multifactorial with vitamin D deficiency, malnutrition, inflammation and chronic therapies being implicated. While respiratory function remains the main focus in CF, CFBD should not be ignored as some studies have reported that over 50% of patients with late stage CF may have osteoporosis. This in turn can lead to a greater risk of developing vertebral compression fractures which are known to be associated with worsening respiratory function [8]. Therefore, early diagnosis and appropriate management of CFBD is crucial in improving the overall health of CF patients.

Numerous studies have reported that BMD can be accurately estimated from computed tomography (CT) scan data with the most widely reported metric being trabecular bone attenuation in lumbar vertebrae [10]. In our institution, an ultra-low dose CT (ULDCT) of the thorax with a dose equivalent to approximately two frontal chest radiographs is used as standard for routine follow-up of CF patients [11]. While a few studies have reported accurate estimation of bone mineral density using thoracic vertebrae trabecular bone attenuation, to our knowledge, this has not been done with ULDCT in a CF specific population. We therefore aimed to explore whether opportunistic screening of CF patients who had undergone routine ULDCT as part of follow-up, would assist in the early diagnosis of osteoporosis in this vulnerable cohort.

## Methods

### Study design and population

Following institutional ethical board approval (Reference: ECM 4 (h) 07/12/2021 & ECM 3 (g) 19/11/2024), a retrospective cohort study was conducted. The study population consisting of one hundred and sixteen cystic fibrosis patients who had ultra-low dose thoracic CT and a DEXA scan within a 12-month period, were identified, and selected from an internal Cystic Fibrosis Registry. Patient demographics including age and sex as well as DEXA scan results were collected for all identified patients. DEXA data collected included: Z-scores and T-scores of the proximal femur (PF) (lowest value of femoral neck or total hip) and lumbar spine (LS) (mean of L1-L4) and BMD (g/cm^2^) of the proximal femur (PF) (lowest value of the femoral neck or total hip) and lumbar spine (mean of L1-L4). Additional 100 random non-contrast, regular dose CT scans of non CF patients within three age groups (18–30, 30–50, 50–75) were collected from the picture archiving communicating system (PACS) for validation of BMD measurements on ULDCT. The non-CF cohort of patients were aged matched and sex matched to our CF cohort to ensure a representative sample was analysed.

### Dual Energy X-ray Absorptiometry (DEXA)

All studies were obtained on the Lunar Prodigy Advance (GE Healthcare, GE Medical Systems, Milwaukee, WI, USA) with either thin mode or standard mode for both the femur and lumbar spine. For the spine, the following settings were used: standard Mode (13–25 cm)—76 kV, 3 mA and 43-s time, thin mode (less than 13 cm) – 76 kV, 0.750 mA and 43-s time. For the femur, the following settings were used: standard mode—76 kV, 3 mA and 44-s time, thin mode – 76 kV, 0.750 mA and 44-s time. A Caucasian population was used as the reference for Z and T score calculations.

DEXA data collected included: Z-scores and T-scores of the proximal femur (PF) (lowest of femoral neck or total hip) and lumbar spine (LS) (mean of L1-L4) and BMD (g/cm^2^) at the proximal femur (PF) (lowest of femoral neck or total hip) and lumbar spine (mean of L1-L4). Osteoporosis, osteopenia and normal BMD was defined as per the WHO criteria. Men under age 50 and pre-menopausal women (assumed to be women < 50), with a Z-score < −2 were classified as having bone mineral density below the expect range and the scan deemed “positive”. In men above the age of 50 and post-menopausal women (assumed to be women ≥ 50) the T-score was used instead where T-score < −2.5 was classified as osteoporotic, T-score between − 2.5 and − 1 as osteopenic and T-score > −1 as normal BMD. For the purposes of categorisation, patients classified as osteoporotic were deemed to have a “positive” scan, while those classified as either osteopenic or normal were deemed to have a “negative” scan.

### CT imaging technique

All studies prior to September 2023 were acquired using a 64-slice multidetector CT scanner (Discovery CT 750 HD; GE Healthcare, GE Medical Systems, Milwaukee, WI, USA) without intravenous contrast material. Following September 2023, all studies were acquired on a 256-slice multidetector CT scanner (Revolution Apex; GE Healthcare, GE Medical Systems, Milwaukee, WI, USA). Our previously published low-dose volumetric protocol was used in all patients [11]. In brief, this acquisition which delivered an effective dose of 0.08 mSv had a tube voltage: 80 kV; tube current: 20 mA; gantry rotation time: 0.4 s; pitch factor: 1.375; and FOV of 32 cm using the Discovery CT 750 scanner while the Revolution Apex scanner used the following parameters: tube voltage: 100 kV; tube current: 30 mA; gantry rotation time: 0.35 s; pitch factor: 1.375 and FOV of 40 cm. Scanning was performed at end-inspiration from lung apices to bases, including costophrenic recesses. No additional expiratory phase imaging was performed. Images were acquired at slice thickness of 0.625 mm and reconstructed at final slice thickness of 3 mm with pure IR (MBIR) in axial, coronal, and sagittal planes (labelled LD-MBIR) and hybrid IR (40% ASIR and 60% filtered back projection) with the Discovery 750 HD scanner. With the Revolution apex scanner, images were again acquired at a slice thickness of 0.625 mm and reconstructed at a final slice thickness of 3 mm with 60% ASIR and high level deep learning.

### Image analysis

Identified CT studies were analysed directly on a dedicated PACS computer by two independent reviewers. Trabecular bone density in Hounsfield Units (HU) was measured at the T4, T7 and T10 vertebrae using the custom shape ROI tool within the PACS (AGFA HealthCare Enterprise Imaging v8.2.0.160) system. The ROIs were measured at the level of the neural foramen and drawn as large as possible without intersecting cortical bone all the while avoiding bone islands, venous plexus, or focal sclerotic or lytic lesions. In addition, patients with vertebral fractures at the vertebrae of interest (T4, T7, T10) were excluded from the study. Patients with incomplete imaging of the T4 or T10 vertebrae were also excluded. Intraclass correlation coefficient was calculated between the two reviewers using a 2-way random model with agreement.

### Statistical analysis

Statistical analysis was carried out using Jamovi (version 2.3.21.0), IBM® SPSS Statistics (version 29.0.1.0) and R (version 4.4). DEXA scan results (Z-scores, T-scores and BMD at PF and LS) were correlated with the average HU values of T4, T7 and T10 vertebrae (T-BD). Multivariate logistic regression was used to develop a formula to predict BMD values in g/cm^2^ of the PF and LS from the average thoracic vertebrae trabecular bone density on CT (measured in HU). Receiver-Operating Characteristic (ROC) curve analysis on the entire cohort with k-fold cross validation was used to develop a threshold HU value below which a positive DEXA scan would be likely in our patient population. For continuous variables, Shapiro–Wilk normality testing directed the use of parametric or non-parametric paired analyses respectively. Levene’s test of variance was used to determine the use of Fischer’s or Welche’s ANOVA as well as guiding the post hoc testing with Games-Howell or Tukey. Mann–Whitney *U* testing was conducted when comparing 2 continuous variables that were not normally distributed but with equal variance. Differences with a *p-*value < 0.05 were considered significant.

### Development of BMD prediction formula

Using multivariate logistic regression, a formula was developed to predict PF and LS BMD values. 69 patients, selected at random, were used as the training set in the development of this formula, while the remaining 45 patients were used to test the formula. Gender and T-BD were found to have a statistically significant correlation with PF BMD and LS BMD and therefore the following formula was created utilising both Gender and T-BD in the prediction of BMD:

$${}_{Predicted}BMD\;=\;\beta0+\left(\beta1\times HU\right)+\left(\beta2\times G\right)$$

β0 = Intercept, β1 = co-efficient representing the relationship between BMD and T-BD, β2 = co-efficient representing the relationship between BMD and gender. G = 1 when gender is male and 0 when gender is female.

Using the above formula, predicted BMD of the PF and LS were calculated and correlation made with actual BMD values as reported on the corresponding DEXA scans to calculate accuracy of the prediction.

Percentage difference was calculated as:

$$Percentage\;difference\;=\;\left[\left(Actual\;BMD-{}_{Predicted}BMD\right)/Actual\;BMD\right]\times100$$

## Results

### Demographics of CF population

Of the CF population, 52 female patients and 62 male patients with mean ages of 34.4 and 35.1 and standard deviations (SD) 12.1 and 11.4 respectively were included in this study.

In this cohort, the mean BMD of the LS was 1.070 g/cm^2^ in females and 1.11 g/cm^2^ in males with no statistically significant difference between the two (*p* = 0.153). The mean BMD of the PF was 0.876 g/cm^2^ in females and 0.967 g/cm^2^ in males with males having a statistically significantly greater BMD of the hip on average than females (*p* < 0.001).

Mean T-BD (HU) for the entire cohort was 210 +/− 41 HU for females and 210 +/− 46.6 HU for males with no statistically significant difference between the two (*p* = 0.925). Inter-Class Correlation between the two readers showed excellent agreement in a 2-way random model at 0.955 (CI: 0.869–0.985).

### Relationship between CT thoracic bone density and DEXA results

**Fig. 1:**
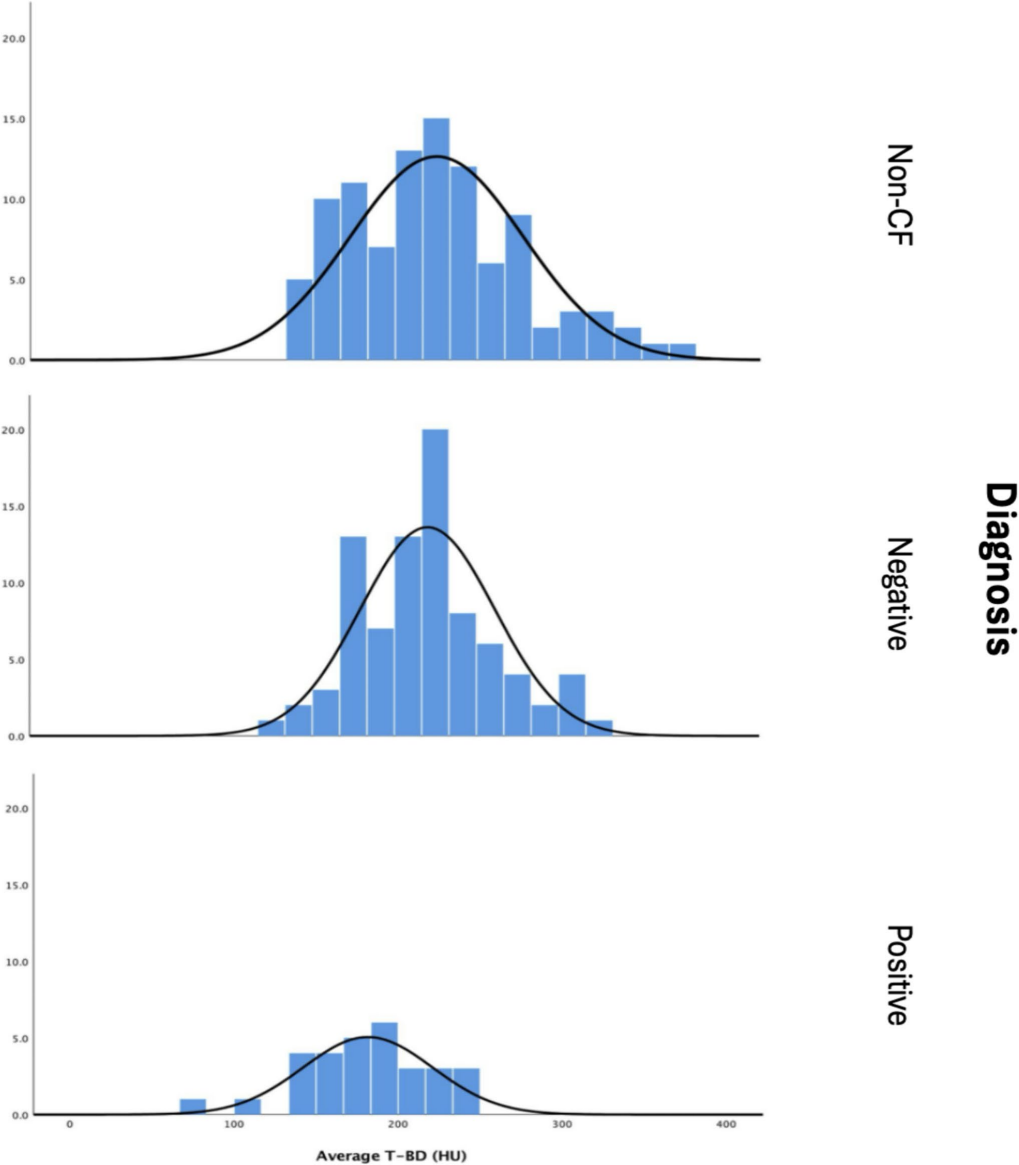
Distribution of T-BD according to diagnosis

DEXA was negative (i.e. normal BMD or osteopenic) in 84/114 (73.7%) patients, and positive (i.e. osteoporotic or BMD below expected for age) in the remaining 30/114 (26.3%) (Table 1). Although there was minor overlap in T-BD (HU) between the negative and positive scans, there was a clear trend to higher values in patients with a negative scan. Those classified as having a negative scan had a mean T-BD of 220 +/− 41HU in comparison to those with a positive scan who had a mean T-BD of 182 +/− 39.5HU (*p* < 0.001). (Fig. 1, Table 2).

**Table 1:** Negative and positive scans in the CF patient cohort

Cohort	Negative (*n* = 84)	Positive (*n* = 30)
*Men* < *50*	42 (36.8%)	14 (12.3%)
*Women* < *50*	31 (27.19%)	14 (12.28%)
*Men* ≥ *50*	5 (4.4%)	1 (1%)
*Women* ≥ *50*	6 (5.3%)	1 (1%)

Where *n*, number of patients; %, percentage of patients

**Table 2:** Relationship between T-BD of CF patients and DEXA diagnosis

	Negative Scan (84, 73.7%)	Positive Scan (30, 26.3%)	MD	*p-value*
T-BD (HU)	220 (41.0)	182 (39.5)	38.6	< 0.001

Where *n*, number of patients; %, percentage of patients; *MD*, mean difference. T−BD is presented as mean with standard deviation in brackets

Comparison was also made between the negative DEXA scan CF cohort and an age matched sample of one hundred non-CF patients who had standard dose, non-contrast CT scans to ensure validity of ULDCT for bone mineral density prediction (Fig. 1). Mann–Whitney *U* testing revealed no statistically significant difference between the two groups (mean difference: − 5.04, *p* = 0.476) thus confirming the non-inferiority of ULDCT for estimating bone mineral density.

Moderately strong correlation was found between T-BD (HU) and BMD (g/cm^2^) of both the LS (*r* = 0.629, *p* < 0.001) and PF (*r* = 0.649, *p* < 0.001). Subgroup analysis found the strongest correlation between T-BD and BMD of the PF to be in males under the age of 50 (*n* = 56, *r* = 0.818, *p* < 0.001) suggesting T-BD as a very accurate measure of BMD of the PF in this cohort. Females under the age of 50, although had a statistically significant correlation between T-BD and BMD of the PF, had only a moderate correlation (*n* = 45, *r* = 0.590, *p* < 0.001) (Table 3).

**Table 3:** Correlation between BMD and T-BD

Group	Number of patients (*n*)	*r *value (PF)	*p *value (PF)	*r *value (LS)	*p *value (LS)
Entire Cohort	114	**0.649**	< 0.001	**0.629**	< 0.001
All Males	62	**0.795**	< 0.001	**0.671**	< 0.001
All Females	52	**0.457**	< 0.001	**0.587**	< 0.001
Males < 50	56	**0.818**	< 0.001	**0.651**	< 0.001
Females < 50	45	**0.590**	< 0.001	**0.622**	< 0.001
Males ≥ 50	6	0.645	0.167	**0.850**	0.032
Females ≥ 50	7	**0.530**	< 0.001	**0.584**	< 0.001

Where *n*, number of patients; *PF*, proximal femur; *LS*, lumbar spine. Boldface *r *values indicate correlations which are statistically significant

### BMD prediction

The relationships varied depending on whether the BMD of the PF or LS was being measured and therefore two formulas were developed using the method described previously:

$${}_{Prediction}BMD_{PF}=\left(0.39596\right)+\left(0.00218\times T-BD\right)+\left(0.118\times G\right)$$

$${}_{Prediction}BMD_{LS}=\left(0.58508\right)+\left(0.00224\times T-BD\right)+\left(0.04823\times G\right)$$

BMD of the LS was calculated with a mean accuracy of +/− 7.76% and a SD of 5.95% while BMD of the PF was calculated with a mean accuracy of +/− 10.07% and a SD of 7.97% (Table 4).

**Table 4:** Accuracy of formula in prediction of BMD on test cohort (*n* = 46)

Predicted BMD	Mean difference (%)	Median difference (%)	Standard Deviation of difference (%)	90th Percentile difference (%)
Proximal Femur	10.07	8.75	7.97	19.36
Lumbar Spine	7.76	6.36	5.95	14.66

Values are represented as percentages

### Prediction of osteoporosis

**Fig. 2:**
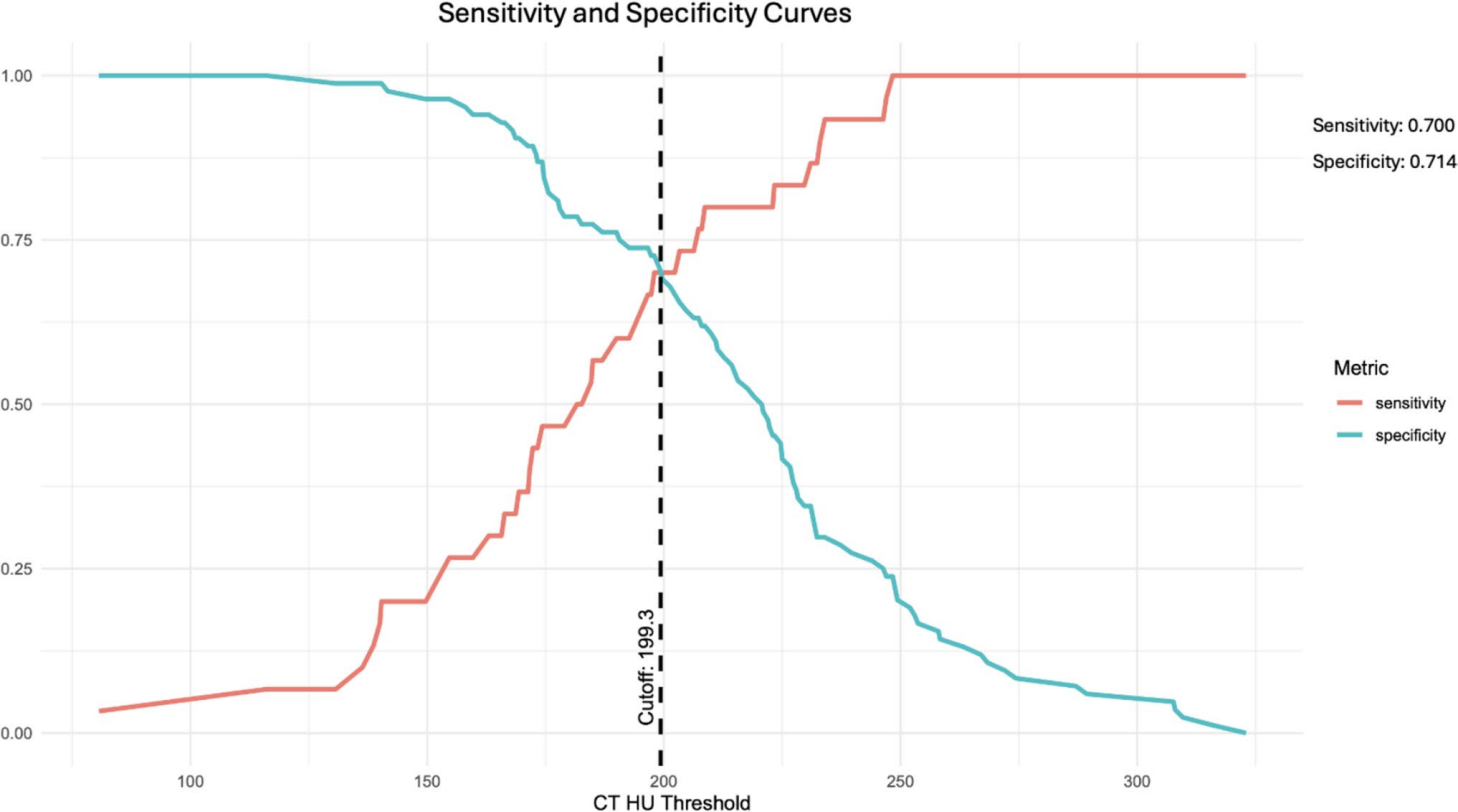
Sensitivity and specificity of osteoporosis prediction based on T-BD

A binary division of the patient cohort into “positive” and “negative” groups was performed with the negative group containing both normal BMD and osteopenic patients as described previously. ROC curve analysis was conducted which found an area under the curve (AUC) of 0.746 and a sensitivity and specificity of 0.700 and 0.714 respectively at a threshold of 199.33HU for the prediction of a positive scan (Fig. 2). Fivefold cross validation was applied which resulted in a mean accuracy of 0.711 (+/− 0.0705), mean sensitivity of 0.7 (+/− 0.139) and mean specificity of 0.715 (+/− 0.112).

## Discussion

This study explored the relationship between T-BD measured on ULDCT thorax and bone mineral density as calculated via DEXA scan in a cohort of cystic fibrosis patients. We aimed to investigate the potential to opportunistically utilise ULDCT thorax to provide additional information on bone health to aid in the diagnosis of CFBD.

Multiple previous studies have examined the relationship between vertebral attenuation on CT and bone mineral density on DEXA with CT of lumbar spine and abdominal CT having been most frequently investigated [10, 12–15]. Although, attenuation on thoracic CT has been less commonly studied in this manner, existing research suggests similar results to lumbar spine vertebral attenuation [16–18]. Kim et al. specifically examined low-dose CT thorax measured vertebral bone attenuation and found a strong correlation (*r* = 0.726, *p* < 0.005) with DEXA [18]. Their research studied a sample of the general population undergoing health screening, however a gap in the literature exists looking specifically at the cystic fibrosis population. We had the advantage of basing our study cohort in a national cystic fibrosis centre in a country with the highest rate of CF per capita globally, providing a large sample of CF patients for inclusion in our study [19].

Several key findings emerged from this study. Firstly, we found a moderately strong correlation between trabecular vertebral bone density measured on ULDCT and BMD measured by DEXA, both in LS and PF. The statistically significant correlation identified in this study is in keeping with previous research and supports the hypothesis that ULDCT could be opportunistically utilised to provide information on BMD in patients with cystic fibrosis.

Interestingly, a significant difference in the degree of correlation was seen between genders. The correlation between T-BD and BMD was strongest in males under the age of 50, with a correlation coefficient of *r* = 0.818 for the proximal femur (*p* < 0.001). This finding suggests that T-BD is an excellent predictor of femoral neck BMD in younger males. The correlation between T-BD and BMD of the PF was weaker in females under 50 (*r* = 0.590, *p* < 0.001). This difference in correlation between genders could be due to the more stable and homogenous bone density patterns observed in males compared to females before the onset of age-related bone loss, as well as the lesser influence of hormonal factors affecting females [20].

Based on the study results, logistic regression was used to develop a formula to predict BMD of the LS and PF on ULDCT with a mean accuracy of +/− 7.76% [SD 5.95%] and +/− 10.07% [SD 7.97%] respectively. Although relatively good accuracy was observed, particularly at the lumbar spine, there exists a margin of error which suggests that it may be more suitable for use in screening or initial assessment of bone health. Future research should aim to refine accuracy by including further variables or increasing sample size.

ROC curve analysis indicated a moderately strong predictive power (AUC = 0.746) for T-BD in diagnosing osteoporosis as has been described in previous research [13, 18]. We identified a threshold for prediction of osteoporosis of 199.33 HU with sensitivity and specificity of 0.700 and 0.714 respectively. Pickhardt et al. describe a significantly lower threshold (135 HU, 0.76 sensitivity, 0.75 specificity) as do Romme et al. (147HU, sensitivity 0.93, specificity 0.97), and Buckens et al. (104HU, sensitivity 0.65, specificity 0.79) [14, 16, 18]. These discrepancies may be explained by differing factors across the various studies. Firstly patient population; our cohort is unique in that it consists solely of CF patients whose disease process may produce differences in bone structure when compared to other populations. In addition, our population consists of a significantly younger cohort with a mean age of 35 in comparison to mean ages of 59.5, 65.9 and 57.9 in the aforementioned studies. Finally, anatomical site of imaging may alter thresholds; our study examines solely thoracic vertebral attenuation whilst most others examine lumbar vertebrae.

While the use of T-BD for predicting osteoporosis shows promise, the moderate sensitivity and specificity values highlight that this method may not be fully reliable as a standalone diagnostic tool. However, ULDCT presents a valuable screening tool, and could potentially be used to identify patients who are at high risk of osteoporosis to prompt further screening with DEXA. The methods which we describe for calculating bone attenuation in HU on CT using an ROI tool require little training, can be easily employed, incur no additional cost and can be conducted without exposing the patient to any further radiation. The use of ULDCT to opportunistically screen for bone disease would contribute to resource stewardship by making efficient and thoughtful use of an existing healthcare resource and minimising waste whilst still delivering high quality care.

Our study had several strengths. The study population was drawn from a well-maintained CF database in a national CF centre, which allowed us to include a relatively large sample of CF patients. Full datasets were available for all patients making it high quality data. The sole inclusion of ULDCT provides a novel angle which has not been largely explored in the evaluation of bone density previously. Another strength lies in the fact that this is an unique and important population whose increased risk of bone disease means that this study fills a valuable gap in the research which mostly examines the general population. That being said, as ULDCT thorax becomes more common and is being utilised in other pathologies such a lung cancer screening, the results from this study may be useful in other populations [21].

Some weaknesses of our study include the relatively small proportion of positive DEXA scans in our cohort. Further studies using a larger sample size may improve this aspect. Another weakness lies in the fact that CT scans and corresponding DEXA scan were conducted up to 12 months apart, allowing for potential changes in bone density and introducing measurement bias. Future studies might consider reducing this time frame to improve accuracy.

This study presents several indications for future research. Larger, prospective studies would be useful to better validate the ULDCT in predicting bone mineral density and diagnosing osteoporosis. This would also allow synchronization of CT and DEXA timing which would improve accuracy. The difference in correlation between T-BD and BMD in males versus females suggests the need for further research with gender-specific analysis to refine prediction models. There is also scope to explore additional clinical markers and integrate fracture risk assessment tools into future studies.

## Conclusion

This study demonstrates ultra-low-dose CT thorax to be a valuable tool for assessing bone mineral density in CF patients, showing a moderately strong correlation between CT-derived bone density and BMD on DEXA. The development of a predictive formula based on CT-derived T-BD and gender offers a practical approach to estimating BMD with reasonable accuracy, particularly in the lumbar spine. A threshold of 199.33HU was identified for prediction of osteoporosis with reasonable sensitivity and specificity. While promising, ULDCT alone may not yet serve as a standalone diagnostic tool for osteoporosis/bone mineral density below expected range for age. Although further research is require to refine accuracy, our study supports the use of ULDCT as a screening tool for bone health in the CF population, encouraging resource stewardship and potentially reducing unnecessary radiation exposure without sacrificing diagnostic value.
